# *Leclercia adecarboxylata* From Human Gut Flora Carries *mcr-4.3* and *bla*_IMP-4_-Bearing Plasmids

**DOI:** 10.3389/fmicb.2019.02805

**Published:** 2019-12-05

**Authors:** Qiaoling Sun, Hanyu Wang, Lingbin Shu, Ning Dong, Fan Yang, Hongwei Zhou, Sheng Chen, Rong Zhang

**Affiliations:** ^1^Department of Clinical Laboratory, Second Affiliated Hospital of Zhejiang University, School of Medicine, Hangzhou, China; ^2^University of Conneticut, Mansfield, CT, United States; ^3^Shenzhen Key Laboratory for Food Biological Safety Control, Food Safety and Technology Research Center, Hong Kong PolyU Shenzhen Research Institute, Shenzhen, China; ^4^State Key Laboratory of Chirosciences, Department of Applied Biology and Chemical Technology, The Hong Kong Polytechnic University, Kowloon, China; ^5^Department of Biophysics and Kidney Disease Center, The First Affiliated Hospital, Institute of Neuroscience, National Health Commission and Chinese Academy of Medical Sciences Key Laboratory of Medical Neurobiology, Zhejiang University School of Medicine, Hangzhou, China

**Keywords:** *Leclercia adecarboxylata*, *mcr-4.3*, gut flora, carbapenem resistance, colistin-susceptibility

## Abstract

A clinical *Leclercia adecarboxylata* strain harboring the *mcr-4.3* and *bla*_IMP-4_ genes was isolated from active rectal screening of carbapenem-resistant Enterobacteriaceae (CRE) in a patient. The isolate was found to harbor seven plasmids, including a 94,635 bp *bla*_IMP-4_-bearing IncN plasmid and a 9,782 bp *mcr-4.3*-bearing ColE10-type plasmid. The isolate was susceptible to colistin despite carrying the *mcr-4.3* gene, suggesting that this MCR-4 variant may not be functional. Carriage of antibiotic resistance genes in human gut *L. adecarboxylata* strain suggests that close surveillance of resistance strains in the human gut flora should be included as a routine clinical practice to prevent occurrence of infections, especially among immunocompromised patients.

## Introduction

Carbapenems are first-line antimicrobial agents used in treatment of multidrug-resistant Gram-negative bacterial infections. However, carbapenem-resistant Enterobacteriaceae (CRE) has emerged as a major public health threat by causing serious infections for which therapeutic choices are limited, with mortality rate as high as 26–44% (Falagas et al., 2014). The prevalence of carbapenem resistance in Enterobacteriaceae is mediated by the rapidly increasing prevalence of carriage of carbapenemase genes. A previous study on nationwide surveillance of clinical CRE strains in China indicated that the *bla*_KPC-2_ and *bla*_NDM_ genes were responsible for phenotypic resistance in 90% of the CRE strains, whereas the *bla*_IMP_ was found in only 3% of CRE strains (Zhang et al., 2017).

Colistin, on the other hand, has been recognized as a last-line agent for treatment of multidrug-resistant Gram-negative bacterial infections, especially for severe infections caused by CRE. However, the emergence of the plasmid-borne colistin resistance gene *mcr-1* in China in 2015 has posed a great challenge to the efficacy of colistin. The product of the *mcr-1* gene is known to modify the phosphoethanolamine moiety of lipid A in the bacterial outer membrane, rendering colistin binding ineffective (Liu et al., 2016). To date, nine *mcr* homologues, ranging from *mcr-1* to *mcr-*9, have been discovered in bacteria from both animals and human (Carroll et al., 2019).

*Leclercia adecarboxylata* is a Gram-negative bacillus belonging to the Enterobacteriaceae family. It is mainly isolated from environmental or animal specimens but has been recognized as an emerging opportunistic pathogen, with the potential to cause severe infections in immunocompromised patients (Spiegelhauer et al., 2019). Although *L. adecarboxylata* is generally susceptible to the common antibiotics, extended-spectrum β-lactamase (ESBL) and metallo-β-lactamase (MBL)-producing strains have been reported in recent years (Shin et al., 2012; Papagiannitsis et al., 2013; Riazzo et al., 2017).

*L. adecarboxylata* is a member of the normal gut flora in animals (Hess et al., 2008). In recent years, animal gut flora is increasingly being regarded as an important reservoir of drug-resistant organisms, which plays a role in promoting transmission of such strains and causing an increase in prevalence of drug-resistant infections in human. Here we describe the identification of a *bla*_IMP-4_ and *mcr-4.3*-bearing *L. adecarboxylata* strain isolated from the intestine of a patient in China.

## Materials and Methods

### Investigation of the Patient and the Rectal Isolate

A 60-year-old man was admitted to the Neurosurgery Unit for treatment of distortion of commissure in November 2018 after being diagnosed with a benign meningioma in the right frontotemporal lobe. The patient was subjected to active rectal screening for the screening of CRE by the enrichment culture supplemented with meropenem in the first 24 h of admission due to the nosocomial infection management (Shen et al., 2018). Briefly, about 1 g of stool sample was inoculated into 5 ml of Luria-Bertani (LB) broths for enrichment and incubated at 37°C for 18 h. A 10 μl aliquot of the enrichment broth was then spread onto a China Blue Lactose Agar plate supplemented with 0.3 μg/ml meropenem and incubated at 37°C for 18 h. The pure colonies were selected and identified using matrix-assisted laser desorption/ionization time-of-flight mass spectrometry (MALDI-TOF MS) (Bruker Daltonik GmbH, Bremen, Germany). A carbapenem-resistant *L. adecarboxylata* strain, designated as Z96-1, was isolated from the stool sample. During the hospital stay, the patient underwent surgery of resection of meningiomas and was prescribed cefoperazone-sulbactam for infection prevention. No infection occurred during hospitalization of this patient, consistently. Likewise, no carbapenem-resistant isolates were recoverable from clinical samples including blood, sputum, and urine.

### Antimicrobial Susceptibility Testing

Antimicrobial susceptibility testing was performed through the broth microdilution method (CLSI, 2018). The MIC values except for colistin and tigecycline were interpreted according to CLSI guidelines, while the resistance breakpoints for colistin and tigecycline were both 2 μg/ml according to the 2018 EUCAST clinical breakpoint tables (available at: http://www.eucast.org/clinical_breakpoints/).

### Whole-Genome Sequencing and Bioinformatics Analysis

Whole-genome sequencing was conducted to investigate the complete sequences of the plasmids utilizing the Illumina HiSeq X10 platform and Nanopore MinION sequencer platform (Li et al., 2018). Complete plasmid sequences were assembled using Unicycler v0.3.0 and modified through Pilon (v1.22) (Walker et al., 2014; Wick et al., 2017), and then annotated with the RAST tool (Overbeek et al., 2014) and Prokka (Seemann, 2014). Analysis of acquired resistance genes was *via* ResFinder 2.1 (Zankari et al., 2012). Plasmid incompatibility type and mobile elements were determined using the bioinformatics tools available from the Center for Genomic Epidemiology^1^ and IS Finder^2^. The whole-genome sequencing data accession number of *L. adecarboxylata* isolate Z96-1 is SAMN11950933 in BioSample (NCBI).

### Ethics Statement

The study was approved by the Ethics Committee of Second Affiliated Hospital, Zhejiang University School of Medicine (2018-039). The subject gave written informed consent in accordance with the Declaration of Helsinki.

### Biosafety Statement

All concerns related to the safe and appropriate use of human-derived materials and infectious agents were approved by the Institutional Biosafety Committee of Second Affiliated Hospital of Zhejiang University, School of Medicine. All experiments were conducted under the guidelines from the Biological Agent Reference Sheet.

## Results and Discussion

### Antimicrobial Susceptibility

*L. adecarboxylata* isolate Z96-1 was found to exhibit resistance to carbapenems, cephalosporins, and cefoperazone-sulbactam according to results of antimicrobial susceptibility tests (shown in Table 1). The strain was found to remain susceptible to piperacillin-tazobactam, aztreonam, ciprofloxacin, amikacin, tigecycline, and colistin (MIC≤0.5 μg/ml).

**Table 1:** The MIC profile of 15 common antimicrobial agents for *mcr-4.3* and *bla*_IMP-4_-bearing *Leclercia adecarboxylata* strain Z96-1.

Drug classes	Antibiotics	MIC (μg/ml)
Carbapenems	Imipenem	8
	Meropenem	16
	Ertapenem	16
Cephalosporin	Cefmetazole	>128
	Ceftazidime	128
	Cefotaxime	128
	Cefepime	64
β-lactams and β-lactamase Inhibitor	Piperacillin-tazobactam	≤8/4
	Cefoperazone-sulbactam	64/4
	Ceftazidime-avibactam	64/4
Others	Amikacin	≤4
	Ciprofloxacin	≤1
	Tigecycline	≤0.25
	Colistin	≤0.5
	Aztreonam	≤4

### Whole-Genome Sequencing Analysis

Analysis of acquired resistance genes *via* ResFinder 2.1 showed that Z96-1 harbored four antimicrobial resistance genes, which encoded resistance to carbapenems (*bla*_IMP-4_), aminoglycosides [*aac(3)-Ib*], fluoroquinolones (*qnrS1*), phenicol (*catB4*) respectively. Interestingly, a plasmid-borne colistin resistance gene *mcr-4.3* was identified in isolate Z96-1, but the gene did not confer phenotypic resistance.

Isolate Z96-1 was found to carry seven plasmids according to results of hybrid assembly. These include a 94,635 bp *bla*_IMP-4_-bearing IncN plasmid pIMP-Z96-1 (CP040895); a 9,782 bp *mcr-4.3*-bearing ColE10-type plasmid pMCR-Z96-1 (CP040891); a 327,617 bp LN794248.1-like plasmid pZ96-1_1 (CP040888); a novel plasmid pZ96-1_2 (CP040893) of 90,410 bp in length; a 75,205 bp pLEC-5e18-like plasmid pZ96-1_3 (CP040894); a 48,894 bp CP020505-like plasmid pZ96-1_4 (CP040892); and a 5,065 bp pKPC45a-like plasmid pZ96-1_6 (CP040890). The 90,410 bp plasmid pZ96-1_2 encoded the RepB replicase and RelE/ParE family, PemK/MazF family toxin. Except pIMP-Z96-1 and pMCR-Z96-1, neither of other plasmids was found to carry antimicrobial resistant genes.

The *bla*_IMP-4_-harboring plasmid, designated as pIMP-Z96-1, was 94,635 bp in length with an average G + C content of 51.4%, and was found to belong to the IncN incompatibility group. The plasmid pIMP-Z96-1 contained four resistance genes [*bla*_IMP-4,_ *qnrS1*, *catB4,* and *aac(3)-Ib*] but not *mcr-4.3*. Results of plasmid comparison *via* BLASTn showed that pIMP-Z96-1 exhibited high sequence homology (>99.9%) but low coverage (<60%) to other known plasmids including *bla*_IMP-4_-harboring plasmid p3 of 52864 bp in length in *Escherichia coli* strain E41-1 (CP028486.1) and the *bla*_NDM-1_-harboring plasmid p4 of 49,215 bp in length in *Klebsiella pneumoniae* strain NUHL30457 (CP026590.1). pIMP-Z96-1 was found to carry several IS*26*, IS*630*, and IS*6100* elements, which might be involved in the evolution by possible plasmid fusions or rearrangements.

**Figure 1:**
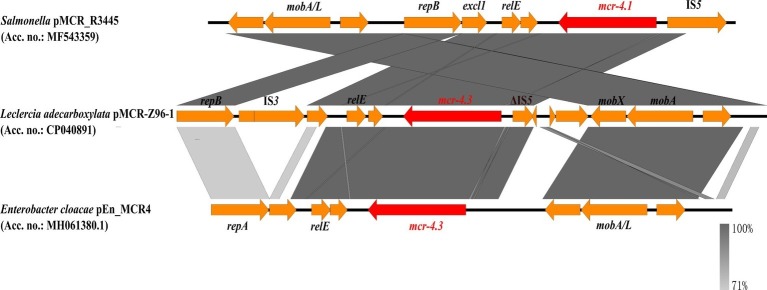
Comparison of the *mcr-4.1*/*mcr-4.3*-neighboring genetic context in the representative plasmids by Easyfig. Colored arrows indicate ORFs and the *mcr-4* genes are highlighted in red. The shaded region depicts sequence similarity. *mcr-4.1* carried by pMCR-R3445 is the prototype for *mcr-4*, whereas *mcr-4.3* of pMCR-Z96-1 and pEn_MCR4 is an inactive variant of *mcr-4* with only two point mutations.

The *mcr-4.3*-harboring plasmid, pMCR-Z96-1, was a 9,782 bp ColE10-type plasmid with an average G + C content of 45.4%. ColE10-type plasmid is known to be compatible with a broad host range and capable of replicating in various bacterial species (Zhang et al., 2019). BLASTn results indicated that the *mcr-4.3*-bearing plasmid pMCR-Z96-1 exhibited 78% query coverage and 99.1% identity to the *mcr-4.1*-bearing plasmid pMCR_R3445 (MF543359), which was isolated from a *Salmonella* strain (Carattoli et al., 2017), but only exhibited 62% query coverage and 98.72% identity to the plasmid pEn_MCR4 (MH061380.1) isolated from a *Enterobacter cloacae* strain which also harbored the *mcr-4.3* gene (Chavda et al., 2018). The *mcr-4.3* gene was found to contain two amino acid substitutions (V179G and V236F) when compared to the *mcr-4.1* located in pMCR_R3445 but exhibited 100% nucleotide identity with the *mcr-4.3* gene in pEn_MCR4. Unlike carriage of the replication gene *repA* in pEn_MCR4, plasmid pMCR-Z96-1 was found to harbor a replication gene *repB* identical to that located in pMCR_R3445 (shown in Figure 1). Genetic environment analysis by Easyfig (Sullivan et al., 2011) revealed that an IS*3* element (nt positions 1031–2118) and an IS*5*-like element (ΔIS*5*, nt positions 5568–5915) were located up- and downstream of *mcr-4.3* in pMCR-Z96-1, which were not present in pMCR_R3445 and pEn_MCR4. This finding revealed that the IS elements (IS*3*, ΔIS*5*) might play an important role in the dissemination of *mcr-4.3*. However, one common region (nt positions 2820–5380) located upstream of the ΔIS*5* element including the *mcr-4* and *relE* genes and one common region (nt positions 6862–8556) located downstream of the ΔIS*5* element including *mobA* and *mobX* genes were shared between pMCR_R3445 and pMCR-Z96-1 (Figure 1). In our study, *mcr-4.3*-bearing *L. adecarboxylata* was susceptible to colistin, which indicated that the *mcr-4.3* gene cannot confer a colistin resistance phenotype. It is possible that *mcr-4.1* gene derived from *mcr-4.3* in chromosomal DNA fragment (~1.7 kb) of *Shewanella frigidimarina* NCIMB 400 (CP000447) (Zhang et al., 2019), and it is also possible that *mcr-4.3* derives from *mcr-4.1* by mutations that silence and likely alleviate the cost of colistin resistance. This observation is consistent with the finding that MCR-4.3 is an inactive form of MCR-4.1, and the two point mutations (V179G and V236F) in MCR-4.3 are responsible for the reduced enzymatic activity in modifying the lipid A, which was already confirmed by the functional expression of two revertant mutants of MCR-4.3 (G179V and F236V) (Chavda et al., 2018; Zhang et al., 2019).

*L. adecarboxylata* is a member of the normal gut flora in animals and has been isolated from human gut (Hess et al., 2008), but attracts little attention. Carriage of antibiotic resistance genes and drug-resistant organisms in the gut flora of human and animals is known to pose a high risk of infection and hence transmission of resistant organisms in hospital settings. The *L. adecarboxylata* strain isolated in this work was found to carry seven plasmids and is therefore likely to serve as a reservoir of antibiotic resistance genes and a medium to disseminate. These findings suggest that close surveillance of resistance strains in the human gut flora should be included as a routine clinical practice to prevent occurrence of infections, especially among immunocompromised patients.

## Data Availability Statement

The datasets generated for this study can be found in the GenBank, SAMN11950933, CP040888-CP040895.

## Ethics Statement

The studies involving human participants were reviewed and approved by the Ethics Committee of Second Affiliated Hospital, Zhejiang University School of Medicine. The patients/participants provided their written informed consent to participate in this study. Written informed consent was obtained from the individual(s) for the publication of any potentially identifiable images or data included in this article.

## Author Contributions

QS and HW did strain characterization and participated in manuscript writing. ND and FY did the whole-genome sequencing. LS and HZ participated in collecting the clinical data and strain characterization. SC participated in the research design, data interpretation, and manuscript writing. RZ designed and supervised the study, interpreted the data, and wrote the manuscript.

### Conflict of Interest

The authors declare that the research was conducted in the absence of any commercial or financial relationships that could be construed as a potential conflict of interest.

## Footnotes

^1^http://genomicepidemiology.org/

^2^https://www-is.biotoul.fr/blast.php
